# Predation risk is associated with head-size-related divergence in breeding phenology in a female sea duck

**DOI:** 10.1007/s00442-026-05891-9

**Published:** 2026-04-24

**Authors:** Bertille Mohring, Ida Hermansson, Kim Jaatinen, Markus Öst

**Affiliations:** 1 Environmental and Marine Biology, Åbo Akademi University, 20500 Turku, Finland; 2 Centre d’Etudes Biologiques de Chizé, UMR 7372 CNRS – Université de La Rochelle, 79360 Villiers-en-Bois, France; 3 School of Environmental Sciences, University of Liverpool, Liverpool, L69 3GP UK; 4 Tvärminne Zoological Station, University of Helsinki, 10900 Hanko, Finland; 5 Finnish Environment Institute, 00790 Helsinki, Finland; 6 Novia University of Applied Sciences, 10600 Ekenäs, Finland

**Keywords:** Brain size, Common eider, Flight initiation distance, Slow–fast life-history continuum, Timing of breeding

## Abstract

Breeding phenological responses to changing environments affect demographics and population persistence. However, inter-individual variation in adjustment of reproductive timing to predation risk has been overlooked. We thus tested whether increasing natural predation risk enhanced or homogenized reproductive timing of females differing in risk sensitivity. Using behavioral (flight initiation distance, FID) and cognitive (relative head volume) proxies of risk sensitivity, we analyzed laying dates of female common eiders (*Somateria mollissima*) breeding in SW Finland (Baltic Sea) relative to conspecifics under fluctuating predation pressure, while accounting for breeding experience and body condition. We found that high predation risk was associated with a divergence of female eider breeding phenologies depending on cognitive but not behavioral proxies of risk sensitivity. Accordingly, relatively large-headed females, assumed to be more risk-sensitive, bred later than small-headed ones following years of high adult or nest predation risk—plausibly reflecting extended nest prospecting in risk-sensitive individuals—leading to greater laying asynchrony. In contrast, females displaying longer FIDs (more risk-sensitive) bred earlier than those displaying shorter FIDs, but this response was irrespective of the level of predation risk. Because FID was measured late in incubation, we hypothesize that it may more strongly reflect state-dependent parental investment in the current breeding attempt than risk sensitivity during nest initiation, with higher parental investment promoting delayed escape. Additionally, experienced breeders bred earlier than inexperienced ones. More attention should be given to cognitive traits and behaviors associated with risk sensitivity to better understand variation in individual breeding phenology and synchrony.

**Supplementary Information:**

The online version contains supplementary material available at 10.1007/s00442-026-05891-9.

## Introduction

Timing of reproduction is a strong determinant of reproductive success (Dunn et al. 2010; Lane et al. 2012) and the ability of organisms to adjust their breeding phenology in response to changing environmental conditions affects demographics and population persistence (Zettlemoyer and DeMarche 2022). Environmental variation (e.g., photoperiod, local climatic conditions, food availability, predation risk; Dunn et al. 2010) affects the timing of reproduction and may promote breeding synchrony given that individuals strive to time their reproduction to match optimal environmental conditions (Ejsmond et al. 2021). However, because intrinsic factors, such as individual state and behavioral traits, also contribute to the timing of reproduction (Verhulst and Nilsson 2008; Abbey-Lee and Dingemanse 2019) and may mediate differential responses to environmental cues (Quinn et al. 2012), individual differences in the timing of reproduction may emerge, producing population-level breeding asynchrony. Importantly, the processes that shape the degree of breeding synchrony are poorly understood but crucial for assessing species vulnerability to contemporary disturbances, such as climate change and anthropogenic alteration of predator–prey relationships (Keogan et al. 2018).

An influential line of research suggests that the timing of breeding relative to conspecifics may serve an antipredator function (Ims 1990). A long-held idea is that synchronous breeding may potentially dilute predation risk on individual nests and/or breeding parents, which is considered particularly relevant in social and colonial species (Rutberg 1987). Nevertheless, because predator densities covary with prey densities via both functional (e.g., changes in feeding rate) and numerical (e.g., changes in predator abundance) responses, asynchronous reproduction may be an optimal antipredator response when predation pressure becomes severe (Ims 1990). Thus, it has become increasingly evident that individual breeding phenology can exhibit plasticity in response to predation risk, and that such plasticity may itself vary among individuals in ways that are evolutionarily relevant (Abbey-Lee and Dingemanse 2019). In that context, the conditions favoring inter-individual variation in reproductive timing in response to fluctuating predation risk remain enigmatic (Ims 1990; Abbey-Lee and Dingemanse 2019).

The emergence of individual variation in responses to predation has been attributed to individual optimisation of risk–reward trade-offs (Sih and Del Giudice 2012; Liedtke and Fromhage 2019). Risk-sensitive individuals are expected to take more time to sample their environment, thus acquiring high-quality information facilitating more accurate decisions (Burns and Rodd 2008; Chittka et al. 2009; Gibelli et al. 2019), hence providing long-term rewards in terms of survival. Conversely, less risk-sensitive individuals strive to maximize short-term rewards by maximizing their current reproductive success, achieved through faster but potentially more error-prone decision-making, associated with potential survival costs (Mathot et al. 2015). The strategy to collect and assess information may thus depend on individual optimisation of cognitive speed–accuracy trade-offs (Sih and Del Giudice 2012)—potentially associated with differences in (relative) brain size (Burns and Rodd 2008)—although the proposed link between behavioral and cognitive responses to risk is controversial (Dougherty and Guillette 2018). Under the risk-reward framework, individual strategies for sampling the environment may also impact inter-individual differences in responsiveness to risk-related cues. Thus, it has been proposed that less risk-sensitive individuals show lower plasticity in their responses to risk and changing environmental conditions, due to their tendency to quickly form inflexible routines (Jones and Godin 2010; Cole and Quinn 2014; Jolles et al. 2019). However, the impact of predation risk on the magnitude of inter-individual differences in risk sensitivity is debated. Thus, increased predation risk has been associated with either homogenized (Cooper and Blumstein 2015; Sommer-Trembo et al. 2016) or diversified (Abbey-Lee and Dingemanse 2019) risk-taking behaviors among individuals. Such contrasting responses could lead to more or less synchronized breeding, with potential benefits in terms of risk dilution and predator avoidance, respectively (Ims 1990). Consequently, the impact of predation risk on individual variation in the timing of breeding and resulting breeding synchrony remains elusive.

Heeding this call, we investigated breeding phenological responses to predation risk in colonially-breeding female common eiders *Somateria mollissima* (hereafter, eiders) in the northern Baltic Sea. The focal population has been subject to rapidly increasing but spatially and temporally variable predation pressure by its native apex predator, the white-tailed eagle *Haliaeetus albicilla* (Öst et al. 2018, 2022), and by mammalian invasive alien predators, American minks *Neogale vison* and raccoon dogs *Nyctereutes procyonoides* (Jaatinen et al. 2022). Our study is based on a comprehensive long-term longitudinal dataset that includes timing of breeding, predation risk, intrinsic state (body condition), as well as behavioral (flight initiation distance, FID) and cognitive (relative head size) proxies of individual risk sensitivity. Indeed, FID is a standardized measure of risk-taking behavior (Frid and Dill 2002; Mohring et al. 2022), and individuals displaying longer FIDs are predicted to be more sensitive to risk than those displaying shorter FIDs and therefore to take more time to explore their environment, also displaying higher plasticity under changing environmental conditions (Dammhahn and Almeling 2012). Similarly, in great tits *Parus major*, individuals with slower exploration behavior—a proxy of risk aversion complementary to, but distinct from, FID—display higher risk sensitivity during reproduction, delaying their return to incubation after a perceived threat (Cole and Quinn 2014). Relative head size is a proxy of relative brain mass in female eiders, as head size is strongly correlated with brain mass with r^2^ = 0.73 (Jaatinen et al. 2019). Individuals with a relatively larger brain size are predicted to have higher cognitive abilities, providing an incentive to acquire higher-quality information facilitating more accurate decisions, and hence to take more time to sample their environment (Kotrschal et al. 2013, 2015; Jaatinen and Öst 2016). This translates into higher risk sensitivity as exemplified by relatively large-headed female eiders, who adjust the speed of antipredator coalition formation according to annual predation risk, unlike small-headed females, who show no such adjustment (Öst and Jaatinen 2015). Parallel findings were obtained in guppies *Poecilia reticulata*, where individuals with larger brains show enhanced predator inspection and spatial memory under predation threat (Burns and Rodd 2008; Kotrschal et al. 2013, 2015). Large-headed female eiders also exhibit higher nest success and survival under severe predation pressure (Jaatinen et al. 2019), likely reflecting more cautious reproductive decisions in risky environments.

We examined how timing of breeding is influenced by FID and relative head size under fluctuating predation risk, controlling for potentially relevant individual quality attributes (breeding experience and body condition). We expected the breeding phenology of phenotypes with different risk sensitivity to either diverge (Abbey-Lee and Dingemanse 2019) or converge (Cooper and Blumstein 2015; Sommer-Trembo et al. 2016) as a response to increasing predation risk.

## Materials and methods

### Study area and species

The field work for this study was conducted on 21 islands in the archipelago around Tvärminne, western Gulf of Finland (59.50°N, 23.15°E), in 2008–2022. The monitoring of the islands was performed from early May to mid-June (range of annual median census dates: 16–23 May).

Eiders are colonial and long-lived sea ducks (average life expectancy: 11.8 years, 95% CI = [5.4–25.2]; Wood et al. 2020), being short-distance migrants (ca 1000 km one-way) in the northern Baltic Sea, with females showing high natal (Coulson 1984; Swennen 1990) and nearly absolute breeding philopatry (Öst et al. 2011). Eiders in the focal population breed in two contrasting habitats: small, open granitic islets with patches of grass and occasional juniper *Juniperus communis* scrub, and larger islands with pine-dominated forest (*Pinus sylvestris*) with interspersed junipers (mean island size (± SD): 2.2 (± 3.0) ha, range: 0.1–10.2 ha; mean forest cover (± SD): 22.3 (± 21.7) %, range 0.0–53.0%). Females lay 3–6 eggs (mean (± SD) annual clutch size in Tvärminne: 4.62 ± 0.17, *n* = 30 years; Öst et al. 2022), incubating the clutch for ca 26 days (Korschgen 1977). Eiders display female-only care, and the precocial ducklings hatch synchronously, leaving the nest within 24 h of hatching (Öst and Bäck 2003). The three most important adult predators, in descending order of importance, are the white-tailed eagle, American mink and raccoon dog (Öst et al. 2018). Importantly, predation on adult eiders in our study system has become increasingly dominated by white-tailed eagles due to effective control of invasive mammalian predators (Jaatinen et al. 2022). This creates a more uniform predation regime that reduces variation in antipredator strategies required for coping with different predator types, thereby simplifying interpretation of antipredator traits in relation to predation risk. The most important egg predators are the hooded crow *Corvus corone cornix* and gulls (*Larus* spp.). The annual proportion of incubating females killed at their nests averages 5.3% (± SD = 4.6%; range 0–16.8%, *n* = 30 years), with a sharply increasing trend (Öst et al. 2022). The vast majority of nest failures are due to egg depredation, as illustrated by data from 2009–2022, during which 90.9% of 2421 nest failures (*n* = 2201) were attributed to predation (Mohring et al. 2023).

### Female breeding phenology and individual traits

Incubating eider females (*n* = 1461 breeding attempts, annual mean 101 ± 34 (SD), range: 42–165; n_ID_ = 475 unique individuals) were captured on the nests with hand nets. The trapping was timed to when most birds had reached the latter part of the incubation period to minimize the probability of abandonment. Laying date was estimated using egg floatation for all active nests, regardless of whether the female was captured. It has been confirmed in this population that the incubation stage as estimated by egg floatation does not differ statistically from the real incubation stage as determined by direct observations of laying or hatching (Kilpi and Lindström 1997). To allow comparison of female laying dates relative to conspecifics and independent of annual phenology, we calculated the relative laying date as the deviation from the annual median laying date of all active nests. We note here that inclusion of untrapped females implies that the mean relative laying date for the captured pool of breeders may not equal zero. The timing of nest monitoring may influence the distribution of observed laying dates on an island. If the monitoring is done early, some late nesters may not have yet initiated incubation, while late monitoring may exclude exceptionally early nesters that have already hatched before the nest visit. We controlled for this potential bias by calculating relative date of the nest visit for each female, expressed as the deviation from the annual median laying date of the whole population (see above) and including it as a covariate in our models (see ‘Statistical analysis’).

Upon capture of an individual, we kept the time of bird handling to a minimum. Each individual female was weighed (with 5 g precision) and the radius-ulna was measured for structural size (with 1 mm precision). Each female was then marked with a standard metal ring and a unique set of color rings to allow later identification. As ducklings are precocial, only adults can be ringed in the study population; birds that we encountered for the first time (no rings) were considered first-time breeders and hence regarded as inexperienced (annual proportion of first-time breeders, mean ± SD: 0.37 ± 0.20, range: 0.03–0.81), whereas recaptured birds (with rings) were categorized as experienced ones. We consider this method of determining breeding experience as reliable for several reasons. First, eiders in the area show high breeding philopatry, the median dispersal distance being only 32.4 m (Hermansson et al. 2026), with island switching remaining infrequent, although it has increased under the current, harsher predation regime (from 1.9% of females switching islands in 1996–2009 to 12.9% in 2003–2023; Öst et al. 2011; Hermansson et al. 2026). In addition to the core study islands—where the study was conducted—female eiders are caught and ringed every year on the surrounding islands, which reduces the risk for the first breeding attempt of the individual to be undetected if island switching occurs. Second, trapping success is high, with the annual proportion of successful captures ranging from 0.55 to 0.71 (Jaatinen et al. 2022).

As eider females fast during incubation, we estimated size-corrected female body condition at hatching, to allow comparison of female body reserves independent of their incubation stage. This index, previously detailed and validated by Öst and Steele (2010), was calculated from standardized residuals of a linear regression of the log-transformed estimated weight at hatching (response variable) against the log-transformed radius–ulna length (independent variable). Weight at hatching was estimated by subtracting the expected daily weight loss for the remainder of incubation from the weight at the time of measuring. The daily weight loss rate was calculated each year as the slope of the linear regression of log-transformed body weight against log-transformed incubation time and projected hatching date (Öst and Steele 2010). Average daily weight loss rate was calculated separately for each year because weight loss in incubating females varies markedly across years (approximately 10–40 g/day; Öst et al. 2008). Body condition at hatching was only estimated for females that had been incubating for at least 5 days, to exclude females that might still be laying eggs (Öst et al. 2022).

We measured female head length, width, and height (with 0.01 mm precision). Head volume, tightly correlated with brain mass in female eiders (Jaatinen et al. 2019), was calculated as their product (with 0.1 cm^3^ precision; within-individual repeatability of head volume measurements across years: *r* ± SE = 0.71 ± 0.02, 95% CI = [0.67, 0.75], *p* < 0.001; ‘rpt’ function, rptR package; Stoffel et al. 2017). As larger females tend to have larger heads (linear regression of log-transformed mean lifetime head volume on log-transformed mean radius–ulna length: mean parameter estimate ± SE: E ± SE = 0.49 ± 0.03, *t* = 18.86, *p* < 0.001, r^2^ = 0.20), we obtained relative head volume by correcting the head volume for radius–ulna length. Relative head volume was calculated as the standardized residuals of a linear regression of the log-transformed head volume (dependent variable) against log-transformed radius–ulna length (independent variable). Mean head measurements and radius–ulna lengths were used in the above regression if repeated measurements were available.

We measured female flight initiation distance with a standardized procedure (Seltmann et al. 2012; Mohring et al. 2022), assuming a negative relationship between FID and risk-taking propensity (Seltmann et al. 2012). FID was measured while revisiting the nests one day before the estimated hatching date. By revisiting each nest at the same phase of incubation, we control for the fact that FID tends to decrease with incubation time due to the increase in brood value (Forbes et al. 1994). FID was measured by first making sure that the female had noticed the researcher before approaching her in a straight line, at a constant speed, and the distance between the researcher and the nest when the female left the nest was measured (to the nearest 10 cm). Given significant repeatability of individual FID in female eiders (within-individual repeatability across years: *r* ± SE = 0.44 ± 0.04, 95% CI = [0.38, 0.52], *p* < 0.001; ‘rpt’ function, rptR package; Stoffel et al. 2017), median lifetime FID was used if repeated FID measurements were available (mean number of FID measurements per individual ± SD = 2.0 ± 1.4, range = 1–9).

### Predation risk

Female eiders possess only limited information about predation risk when initiating nesting. In contrast, the timing of breeding is expected to be influenced by past conditions in long-lived animals (Grieco et al. 2002). For instance, selection should favor a change in breeding site after nest failure (Switzer 1993), typically caused by predation in birds (Lima 2009). A predation-induced increase in breeding dispersal, in turn, may delay breeding in the following year, with breeding delayed by approximately 4–5 days for each additional kilometer of dispersal (Öst et al. 2011). We therefore calculated island-specific predation risk indices using records of depredated nests and adults on the breeding islands in the year preceding a focal individual’s breeding attempt. Island-specific adult predation risk was measured as the number of killed females found in the year preceding the breeding attempt on an island, divided by the yearly number of nests on that island and year. Likewise, island-specific nest predation risk was calculated by dividing the number of depredated nests at the end of the breeding season preceding the breeding attempt by the total number of nests on the island where the breeding attempt took place. The fate of each nest was recorded at revisits either at the estimated hatching date or during a final census. We then documented the presence of ducklings. However, because broods leave the nest within one day after hatching (Öst and Bäck 2003), we examined the state of the eggshells if the brood had already left the nest (Öst and Steele 2010). Only hatched eggshells have a visible leathery membrane, whereas depredated eggs have yolk or blood on the shells. The fate of the great majority of nests (4593 out of 5416 nests, 84.8%) could be classified as either successful (at least one duckling or leathery membrane found) or unsuccessful (depredated eggs or abandoned or infertile clutches). For the unsuccessful nests, predation was the main cause of nest failure (2369 depredated out of 2592 unsuccessful breeding attempts, 91.4%).

### Statistical analysis

Statistical analysis was run in R 4.0.4 (R Core Team 2023). All independent variables were centered and scaled and we checked that the assumptions of normality and homoscedasticity of residuals were met in the models. Given that island-specific adult and nest predation risk were correlated (*r* = 0.42, *p* < 0.01, see Electronic Supplementary Figure S2), we constructed two separate linear mixed models (LMMs, ‘lmer’ function, lme4 package; Bates et al. 2015) with relative laying date as the dependent variable and relative date of nest visit, breeding experience, body condition, relative head volume, median lifetime FID, and either island-specific nest predation risk (Model M_1_) or island-specific adult predation risk (Model M_2_) as independent variables. We specifically tested for the presence of differential breeding phenological responses to predation risk depending on cognitive or behavioral proxies of risk sensitivity. To this end, we included the two-way interactions between island-specific nest predation risk and relative head volume and between island-specific nest predation risk and median lifetime FID (Model M_1_), as well as the corresponding two-way interactions including island-specific adult predation risk and the two proxies of risk sensitivity (Model M_2_). Noteworthy, median lifetime FID and relative head volume were not correlated with each other (*r* = –0.05, *p* = 0.07, see Electronic Supplementary Figure S2). Female and island identity were added as random effects to control for the potential pseudo-replication arising from repeated observations of the same female over the study period and repeated observations of individuals on the same island. We verified the lack of multi-collinearity among the independent variables of the two models (all variance inflation factors were less than 1.17, i.e., well below the conservative threshold of 2.5; Allison 2012). To verify the robustness of our results, we also performed model averaging (‘model.avg’ function, MuMIn package; Barton 2020) according to Akaike's Information Criterion corrected for small sample size (AICc). To this end, all possible combinations of the candidate independent variables and interactions in M_1_—respectively in M_2_—were run using the ‘dredge’ function (MuMIn package; Barton 2020). Model averaging produced qualitatively similar results to the full model (see Electronic Supplementary Tables S1 and S2). We carried out an additional analysis in which we relied on a within-subject centering approach (van de Pol and Wright 2009) to test whether associations with FID reflected consistent among-individual differences or within-individual variability. We fitted versions of models M_1_ and M_2_ in which FID was decomposed into a between-individual component (individual mean FID) and a within-individual component (annual deviation from the individual mean), including their interactions with predation risk. The results supported effects being driven by consistent among-individual differences in FID, reported in the Electronic Supplementary Table S3.

We did not additionally control for median breeding phenology in the analysis, as there is no correlation between the annual median laying date and the synchrony (interquartile range) in laying dates, as reported in the supplementary data of our previous 30-year study (Öst et al. 2022) (r_30_ = 0.22, *p* = 0.25). Furthermore, our use of relative laying dates—centered around the population median laying date—essentially factors out any climate-associated impacts on absolute breeding phenology. Importantly, however, annual population-level breeding synchrony (i.e., interquartile range) is unrelated to climate variables or spring migration phenology in this population (Öst et al. 2022).

## Results

**Fig. 1:**
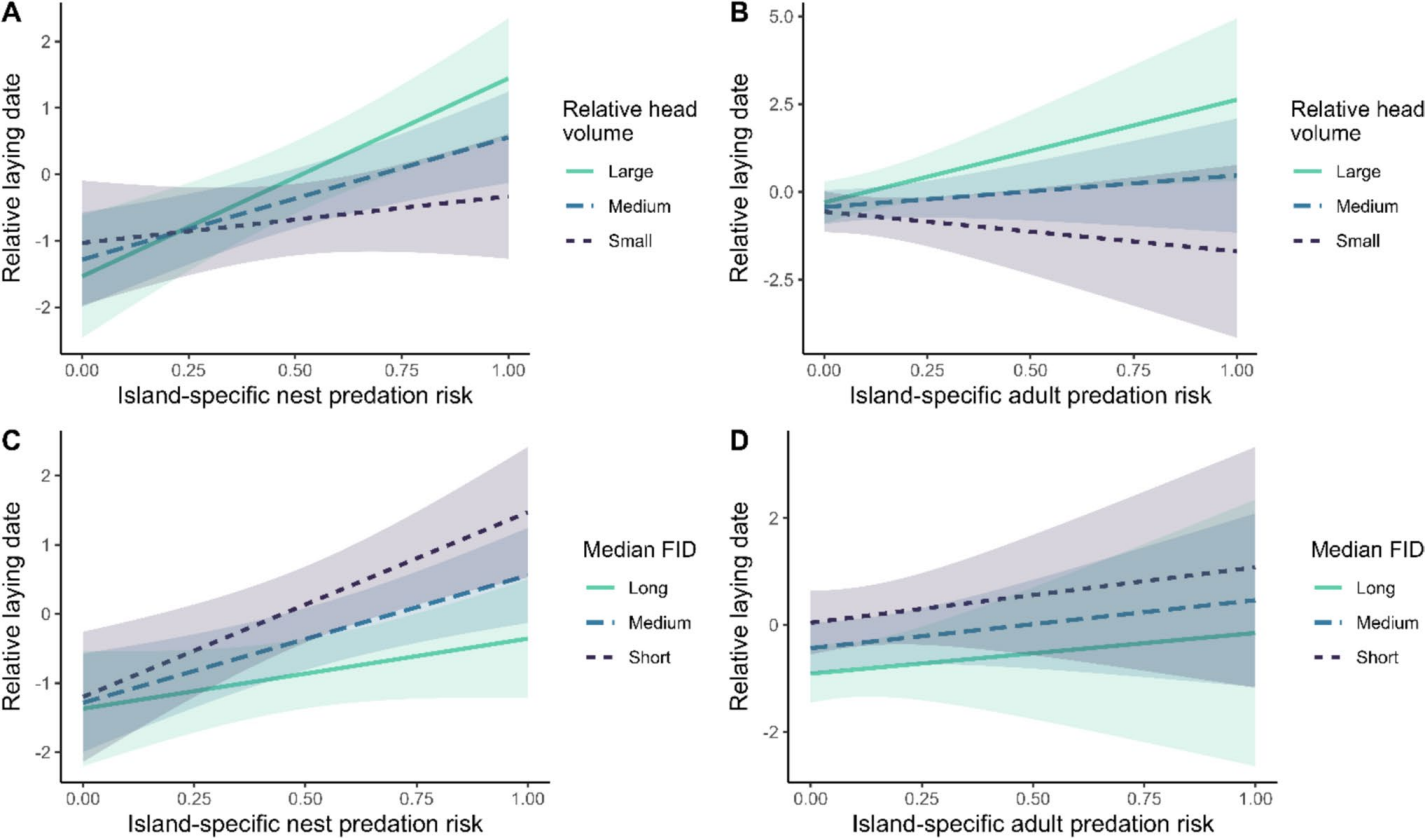
Relationship between female common eider relative laying date and the two-way interactions between (**A**) island-specific nest predation risk and relative head volume, **B** island-specific adult predation risk and relative head volume, **C** island-specific nest predation risk and median lifetime FID and (**D**) island-specific adult predation risk and median lifetime FID. The solid line denotes females with (**A**, **B**) a large relative head volume or (**C**, **D**) a long FID (mean + 1 SD), the dashed line females with (**A**, **B**) an intermediate relative head volume or (**C**, **D**) an intermediate FID (mean) and the dotted line females with (**A**, **B**) a small relative head volume or (**C**, **D**) a short FID (mean—1 SD). Areas account for 95% confidence intervals

**Fig. 2:**
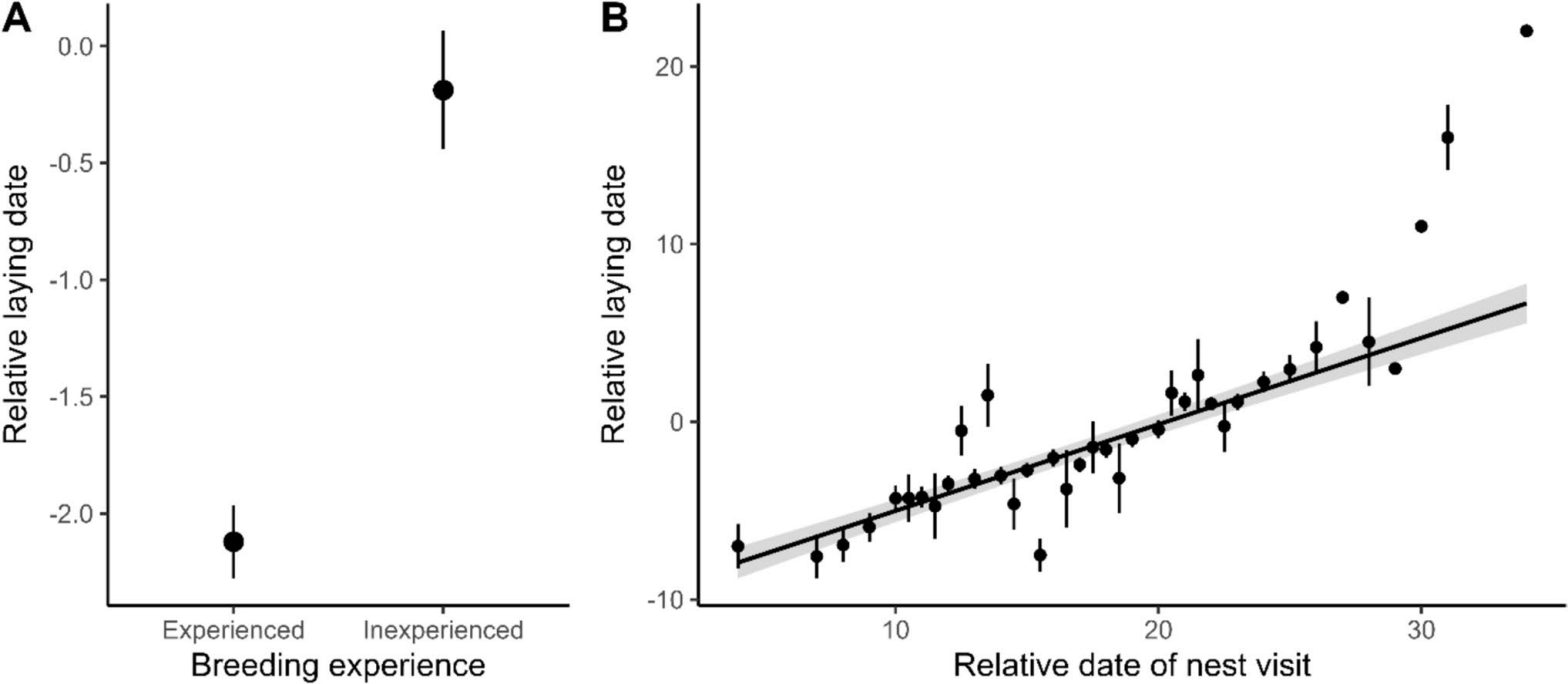
Relationship between female common eider relative laying date and (**A**) female breeding experience (experienced: *n* = 1071, inexperienced: *n* = 390) and (**B**) relative date of nest visit. Black dots correspond to mean values of relative laying dates for (**A**) experienced and inexperienced breeders or (**B**) relative dates of nest visit, and bars account for standard errors. Regression lines depict average fixed effects without integrating random effects and gray areas account for 95% confidence intervals

We found significant two-way interactions between relative head volume and island-specific nest predation risk (Model M_1_; Table 1; Fig. 1A) as well as between relative head volume and island-specific adult predation risk (Model M_2_; Table 1; Fig. 1B) on relative laying dates. In contrast, our results do not provide support for significant two-way interactions between island-specific nest or adult predation risk and median lifetime FID on relative laying dates (nest: *p* = 0. 124, Model M1; adult: p = 0. 929; Model M_2_; Table 1; Fig. 1C, D). Under high nest predation risk, relatively large-headed females initiated breeding later than relatively small-headed ones (Fig. 1A). With regards to adult predation risk, females with a larger relative head size bred even later under increased predation risk, while those with smaller heads advanced their breeding (Fig. 1B). Overall, increasing nest or adult predation risk was thus associated with more pronounced differences in timing of breeding of individuals with different relative head volumes (Fig. 1A and 1B). Females displaying shorter FIDs bred later than those with longer FIDs and there was a similar delay in breeding when nesting on islands with higher nest or adult predation risk, irrespective of female FID (Fig. 1C and 1D)—i.e., independently from behavioral risk sensitivity phenotypes. To aid the visualization of the two-way interaction effects, we only present predicted response slopes and their confidence intervals in Fig. 1, while further description of these interactions (including the raw data) can be found in Electronic Supplementary Figure S1. Furthermore, experienced females bred earlier than inexperienced ones (Table 1, Fig. 2A), and delayed breeding was associated with a late relative date of nest visit (Table 1, Fig. 2B). Lastly, relative laying date was not associated with female body condition (Table 1).

**Table 1:** LMMs explaining female common eider relative laying date in relation to individual characteristics (breeding experience, relative head volume, median lifetime FID), relative date of nest visit, nest (M_1_) or adult (M_2_) predation risk and the two-way interactions between the predation risk indices (nest (M_1_) or adult (M_2_) predation risk) and female relative head volume and median lifetime FID, respectively

Fixed effect	Mean estimate (E) ± SE	t-value	*p*-value
M_1_: Nest predation risk model			
Intercept	−0.40 ± 0.28	−1.42	0.164
Breeding experience: Experienced	**−1.67 ± 0.25**	**−6.56**	** < 0.001**
Body condition	−0.12 ± 0.14	−0.92	0.361
Relative head volume	**0.32 ± 0.15**	**2.07**	**0.039**
Median lifetime FID	**−0.47 ± 0.15**	**−3.24**	**0.001**
Nest predation risk	**0.32 ± 0.12**	**2.65**	**0.008**
Relative date of nest visit	**2.10 ± 0.12**	**17.00**	** < 0.001**
Relative head volume × nest predation risk	**0.25 ± 0.12**	**2.18**	**0.030**
Median FID × nest predation risk	−0.16 ± 0.11	−1.54	0.124
M_2_: Adult predation risk model			
Intercept	−0.36 ± 0.29	−1.24	0.223
Breeding experience: Experienced	**−1.69 ± 0.26**	**−6.62**	** < 0.001**
Body condition	−0.10 ± 0.14	−0.73	0.463
Relative head volume	**0.32 ± 0.15**	**2.10**	**0.037**
Median lifetime FID	**−0.47 ± 0.15**	**−3.18**	**0.002**
Adult predation risk	0.04 ± 0.12	0.33	0.741
Relative date of nest visit	**2.11 ± 0.12**	**17.01**	** < 0.001**
Relative head volume × adult predation risk	**0.26 ± 0.13**	**2.07**	**0.038**
Median FID × adult predation risk	−0.01 ± 0.13	−0.09	0.929

Female and island identity were included as random effects

Significant effects (*p* ≤ 0.05) are presented in bold

## Discussion

We tested whether increasing predation risk is associated with increased or decreased differences in timing of breeding depending on behavioral and/or cognitive sensitivity to risk, for the first time in prey exposed to natural predation risk. Using data on incubating female eiders spanning 15 years, we found that increasing predation risk on both adults and nests was associated with a divergence of breeding phenologies depending on relative head volume, a presumed correlate of risk sensitivity. Thus, relatively large-headed females, presumed to spend more time on nest prospecting (see below), bred significantly later following years of high island-specific adult predation risk while relatively small-headed ones bred significantly earlier. Likewise, the extent of the breeding delay following years of elevated nest predation risk was more pronounced in relatively large-headed females compared to their smaller-headed counterparts. These divergent responses should lead to greater breeding asynchrony as an aftermath of severe predation risk, a finding echoing that of Abbey-Lee and Dingemanse (2019) in great tits nesting under experimentally induced predation risk. To illustrate with concrete numbers, an increase in the per nest probability of breeder mortality of 0.5 (0.25–0.75) corresponds to ca 2 days greater spread in breeding dates of relatively large-headed (mean + 1 SD) and small-headed (mean–1 SD) females in the subsequent season (Fig. 1B). This difference is not trivial given compelling evidence that breeding delays can be costly (Verhulst and Nilsson 2008; Descamps et al. 2011). However, although longer FIDs indicate greater risk sensitivity (Réale et al. 2010), breeding phenological responses to increasing predation risk remained consistent across individuals, despite individual variation in this behavioral trait. We hypothesize that the lack of a differential response may arise from the fact that FID in incubating parents may better reflect differences in parental investment (see below).

Larger brains may be associated with slower but more thorough decision-making (Burns and Rodd 2008; Kotrschal et al. 2013, 2015). This is expected to reflect individual optimisation of cognitive speed–accuracy trade-offs, allowing plastic responses to risk and changing environmental conditions (Sih and Del Giudice 2012). While the relationship between (relative) brain size and breeding phenology is little explored in an avian context, large-headed female red-backed shrike *Lanius collurio* (Bialas et al. 2024) and common eider (Öst and Jaatinen 2015) had a later onset of breeding, consistent with theoretical expectations. In the absence of direct observations of nest prospecting, it remains crucial to assess whether delayed breeding in large-headed females reflects longer habitat sampling before selecting a nest. Indeed, several lines of evidence (see below) suggest that relatively large-headed birds are characterized by a late onset of egg-laying, likely due to thorough nest prospecting. We found that relatively small-headed female eiders had an advanced breeding schedule compared to their larger-headed counterparts across the range of observed predation risk levels, except for the very lowest levels of predation risk (Figs. 1A and 1B). Importantly, relatively large-headed female eiders show a more pronounced breeding delay when dispersing to unfamiliar and hence potentially dangerous nesting areas than their smaller-headed counterparts (Jaatinen and Öst 2016). Nevertheless, brain size is a contentious proxy of cognition (Smaers et al. 2021), highlighting the need to consider alternative explanations. Larger-brained birds may require more time to acquire sufficient resources for reproduction due to the high energetic costs of maintaining brain tissue (Aiello and Wheeler 1995). If food conditions have deteriorated over the study period, this could create spurious associations between relative head size, breeding phenology, and predation risk. Although possible, this explanation is unlikely for several reasons. First, local populations of blue mussels *Mytilus trossulus*, the staple food of eiders, have remained abundant and stable throughout the study period (Jaatinen et al. 2021). Second, annual variation in the arrival-to-laying interval of female eiders is best explained by white-tailed eagle abundance (Öst et al. 2022). Third and most importantly, breeding dispersal, which requires females to explore new areas, delays breeding in female eiders (Öst et al. 2011). It is reasonable to assume that the additional delay observed specifically in relatively large-headed females in years with extended dispersal (Jaatinen and Öst 2016) is a result of more cautious nest-site prospecting, rather than other unlikely scenarios such as complex interrelationships between relative head volume, dispersal distance and, e.g., arrival time at the breeding grounds.

Importantly, we found that increasing predation risk on both adults and nests was associated with a divergence in breeding phenology depending on individual relative head volume. Breeding on islands characterized by elevated nest predation risk correlated with a delay in breeding the following year; however, this delay was pronounced in relatively large-headed females. Notably, relatively large-headed females bred later following high past adult predation risk, whereas smaller-headed ones bred earlier. A plausible explanation for the stronger divergence of breeding phenology in response to adult rather than offspring predation risk could be that individual risk to breeders may provide a stronger incentive for evolving plastic behavioral responses, as long-lived species should prioritize their own survival at the expense of current reproduction (Gaillard and Yoccoz 2003). Accordingly, female eiders plastically adjust their escape responses (FID) to the perceived predation risk on themselves but not on their nests (Mohring et al. 2022). However, alternative explanations for any differential carry-over effects warrant consideration. One of them is the ‘win-stay-lose-shift’ strategy of nest-site selection, where predation induces increased breeding dispersal (Switzer 1993), often delaying breeding in the following season (Blums et al. 2003), as also observed in eiders (Öst et al. 2011). Yet predation in eiders and other waterfowl tends to occur relatively earlier in dangerous years (Brzeziński et al. 2018; Jaatinen et al. 2019), exposing late-breeding, relatively large-brained females to lower predation rates under such conditions (Öst and Jaatinen 2015). Thus, large-brained individuals may show weaker ‘win-stay, lose-shift’ responses than smaller-brained ones under high predation pressure. Consequently, the differential carry-over effect of adult predation risk on breeding phenology likely reflects differences in perceived predation risk linked to brain size, rather than merely reflecting brain-size-related variation in dispersal driven by predation-induced breeding failure. The resultant decrease in breeding synchrony may convey potential survival advantages through predator avoidance, because instantaneous nest densities in the colonies remain lower over the course of the breeding season.

Taken together, these patterns are consistent with a life-history trade-off in which phenological decisions can influence exposure to predation, potentially mediating the balance between current reproduction and adult survival. Though relative head volume was associated with a divergence of breeding phenology under predation risk, some assumptions of Abbey-Lee & Dingemanse’s (2019) model may not hold here. The advanced breeding schedule of less risk-sensitive individuals (i.e., here, relatively small-headed birds) under high predation risk should allow them to avoid the peak in predation intensity (Abbey-Lee and Dingemanse 2019). However, the opposite trend emerges from our previous work. Because predation events occur seasonally earlier, in relative terms, when annual predation is severe (Jaatinen et al. 2019), predation on both adult breeders and nests disproportionately affects early breeders under such conditions (Öst and Jaatinen 2015; Jaatinen et al. 2019).Delaying breeding in risky years could reduce exposure to peak danger even if late breeding carries reproductive costs (e.g., reduced seasonal opportunity for re-nesting or lower offspring value; Hanssen and Erikstad 2013). The fitness benefits of more cautious reproductive decisions in risky environments are reflected in higher survival rate and higher nest success of large-headed females breeding under severe predation pressure (Jaatinen et al. 2019). Selection is therefore expected to favor large-headed females when predation risk is high. Conversely, under relaxed predation pressure, the benefits of early breeding may dominate, conferring reproductive and survival benefits to relatively small-headed females (Öst and Jaatinen 2015; Jaatinen et al. 2019). Given that morphology, including head and brain size, is heritable (Larsson et al. 1997; Logan et al. 2016), variation in relative brain size in eiders may be maintained in part by a fluctuating direction of selection linked to fluctuating predation pressure, potentially favoring small brain size in benign years and large brain size in dangerous ones (Jaatinen et al. 2019). This, in turn, is expected to translate into among-individual variation in breeding phenology. A generalizable test of this idea would be to estimate seasonal predation hazard across the breeding season and ask whether (i) phenotype-dependent breeding phenology predicts both nest success and adult survival and (ii) the direction or strength of selection shifts across years characterized by differential predation pressure and timing of the predation peak. The mismatch between our results and the assumptions of Abbey-Lee and Dingemanse’s (2019) model underscores the need for further research into the fitness consequences of fast and slow reproductive strategies.

Females with shorter FIDs—presumed to be less risk-sensitive—bred later than those with longer FIDs, and behavioral phenotypes were not associated with variation in phenological response to increasing predation danger. This result is not in agreement with the expectation that risk-sensitive individuals—characterized by longer FIDs (Frid and Dill 2002; Stankowich and Blumstein 2005)—should display stronger plastic responses to changes in their environment such as increasing predation pressure (Dammhahn and Almeling 2012). Although longer FIDs are commonly interpreted as higher risk sensitivity, our FID measurements were taken just before hatching, when escape decisions may be strongly shaped by the value of the current clutch and residual reproductive value. Thus, the association between later breeding and shorter FID may reflect state-dependent parental investment and willingness to accept risk at the nest, potentially masking any association with predation risk assessment during the earlier prospecting phase. This interpretation is consistent with evidence that late breeders exhibit shorter FID and habituation over repeated approaches in the same season (Mohring et al. 2025), perhaps because such females place higher value on their current clutch due to lacking re-nesting opportunities, unlike early breeders who may still re-nest (Hanssen and Erikstad 2013). Furthermore, shorter FID is associated with both larger clutch size (i.e., higher current reproductive investment) (Mohring et al. 2025) and older age (Mohring et al. 2022), consistent with increased risk-taking as future reproductive potential declines (Curio 1983). To conclude, we suggest that risk-taking during the nest prospecting phase or direct measures of exploration (cf. Abbey-Lee and Dingemanse 2019) may be preferable to late-incubation FID when studying phenological responses to predation risk in a context of nest-site selection. Experimental tests of whether pre-laying behavior predicts breeding phenological responses to predation risk may be particularly feasible in nest-box breeding passerines, which are more amenable to experimental manipulation than waterfowl. In these species, pre-laying prospecting and exploration can be quantified, and perceived predation risk manipulated before laying via predator cues (e.g., predator calls or models; Zanette et al. 2011; Abbey-Lee and Dingemanse 2019).

We found that experienced females bred earlier than inexperienced ones (Fig. 2A). Because body condition did not influence the relative laying date (also see below), this difference is not a simple outcome of older age classes consisting of higher-quality individuals possessing greater energy reserves, which could facilitate early nest initiation (Descamps et al. 2011). Females with prior breeding experience may be more efficient at accumulating the necessary resources to initiate reproduction (Verhulst and Nilsson 2008) and physiologically more primed for an early breeding onset (Angelier et al. 2007). Furthermore, site familiarity may reduce the time needed for nest prospecting or allow experienced females to locate safe sites with low rates of nest depredation (Öst and Steele 2010), thereby further reinforcing philopatry and early nesting in the subsequent season (Öst et al. 2011).

Surprisingly, female body condition was not associated with relative laying dates. However, the low repeatability of relative laying date (repeatability, *r* = 0.26 ± 0.03, 95% CI = [0.20, 0.31], *p* < 0.001; ‘rpt’ function, rptR package; Stoffel et al. 2017) suggests that individuals can flexibly adjust timing of breeding in response to environmental conditions, with individual quality playing a limited role. Population-level evidence supports this, suggesting that predation risk, rather than body condition, is linked to variation in breeding phenology. If body condition were the main driver, life-history theory predicts reduced breeding synchrony in early breeding years (Ejsmond et al. 2021). Instead, breeding synchrony increases with higher winter North Atlantic Oscillation (NAO) indices, which signal earlier ice break-up and presumably more favorable conditions (Lehikoinen et al. 2006). Furthermore, increasing annual island nest predation risk best predicted the increase in breeding asynchrony over the past 30 years (Öst et al. 2022). These population-level patterns are consistent with our finding that high predation risk is associated with pronounced asynchrony in individual breeding phenologies, which in turn relates to differences in relative brain size (Fig. 1B).

## Conclusion

To conclude, we showed that individualized breeding phenological responses to perceived predation risk were associated with inter-individual variability in breeding phenology under natural predation risk, with implications for population-level breeding synchrony. The most pertinent finding was that scheduling of breeding in response to natural variation in predation risk was contingent on relative brain size. Accordingly, our study highlights the importance of considering cognitive correlates of individual sensitivity to predation threat for a better understanding of the variation in timing of breeding and population-level breeding synchrony. Such endeavors represent crucial steps toward elucidating how individual differences in cognitive traits and breeding phenology contribute to population adaptability and resilience in the face of predation pressure and other environmental changes.

## Supplementary Information

Below is the link to the electronic supplementary material.

Supplementary file1 (DOCX 444 KB)

## Data Availability

The datasets used in this study are available from the corresponding authors on reasonable request.

## References

[CR1] Abbey-Lee RN, Dingemanse NJ (2019) Adaptive individual variation in phenological responses to perceived predation levels. Nat Commun 10(1):1601. 10.1038/s41467-019-09138-5 30962485 10.1038/s41467-019-09138-5 PMC6453887

[CR2] Aiello LC, Wheeler P (1995) The expensive-tissue hypothesis: The brain and the digestive system in human and primate evolution. Curr Anthropol 36:199–221. 10.1086/204350

[CR3] Allison P (2012) When can you safely ignore multicollinearity. Statist Horizons 5:1–2

[CR4] Angelier F, Weimerskirch H, Dano S, Chastel O (2007) Age, experience and reproductive performance in a long-lived bird: A hormonal perspective. Behav Ecol Sociobiol 61:611–621. 10.1007/s00265-006-0290-1

[CR5] Barton K (2020) MuMIn: multi-model inference. R package version 1.43.17. https://CRAN.R-project.org/package=MuMIn

[CR6] Bates D, Mächler M, Bolker B, Walker S (2015) Fitting Linear Mixed-Effects Models Using **lme4**. J Statist Software 67(1):1–48. 10.18637/jss.v067.i01

[CR7] Bialas JT, Dylewski Ł, Tobolka M (2024) Brain size mediates the choice of breeding strategy in the red-backed shrike *Lanius collurio*. Integr Zool 19:683–693. 10.1111/1749-4877.12803 38196090 10.1111/1749-4877.12803

[CR8] Blums P, Nichols JD, Lindberg MS et al (2003) Factors affecting breeding dispersal of European ducks on Engure Marsh Latvia. J Anim Ecol. 10.1046/j.1365-2656.2003.00698.x

[CR9] Brzeziński M, Chibowski P, Gornia J et al (2018) Spatio-temporal variation in nesting success of colonial waterbirds under the impact of a non-native invasive predator. Oecologia 188:1037–1047. 10.1007/s00442-018-4270-8 30317388 10.1007/s00442-018-4270-8 PMC6244866

[CR10] Burns JG, Rodd FH (2008) Hastiness, brain size and predation regime affect the performance of wild guppies in a spatial memory task. Anim Behav 76:911–922. 10.1016/j.anbehav.2008.02.017

[CR11] Chittka L, Skorupski P, Raine NE (2009) Speed–accuracy tradeoffs in animal decision making. Trends Ecol Evol 24:400–407. 10.1016/j.tree.2009.02.010 19409649 10.1016/j.tree.2009.02.010

[CR12] Cole EF, Quinn JL (2014) Shy birds play it safe: Personality in captivity predicts risk responsiveness during reproduction in the wild. Biol Lett 10:20140178. 10.1098/rsbl.2014.0178 24829251 10.1098/rsbl.2014.0178 PMC4046374

[CR13] Cooper WE, Blumstein DT (2015) Escaping from predators: an integrative view of escape decisions. Cambridge University Press

[CR14] Coulson J (1984) The population dynamics of the Eider duck *Somateria mollissima* and evidence of extensive non-breeding by adult ducks. Ibis 126:525–543. 10.1111/j.1474-919X.1984.tb02078.x

[CR15] Curio E (1983) Why do young birds reproduce less well? Ibis 125:400–404. 10.1111/j.1474-919X.1983.tb03130.x

[CR16] Dammhahn M, Almeling L (2012) Is risk taking during foraging a personality trait? A field test for cross-context consistency in boldness. Anim Behav 84:1131–1139. 10.1016/j.anbehav.2012.08.014

[CR17] Descamps S, Bêty J, Love OP, Gilchrist HG (2011) Individual optimization of reproduction in a long-lived migratory bird: a test of the condition-dependent model of laying date and clutch size: optimal reproduction in a long-lived bird. Funct Ecol 25:671–681. 10.1111/j.1365-2435.2010.01824.x

[CR18] Dougherty LR, Guillette LM (2018) Linking personality and cognition: a meta-analysis. Phil Trans R Soc B 373:20170282. 10.1098/rstb.2017.0282 30104427 10.1098/rstb.2017.0282 PMC6107561

[CR19] Dunn PO, Winkler DW, Møller AP et al (2010) Effects of climate change on timing of breeding and reproductive success in birds. Effects Climate Change Birds 11:113–128

[CR20] Ejsmond A, Forchhammer M, Varpe Ø et al (2021) Nesting synchrony and clutch size in migratory birds: capital versus income breeding determines responses to variable spring onset. Am Nat 198:E122–E135. 10.1086/716064 34559609 10.1086/716064

[CR21] Forbes MRL, Clark RG, Weatherhead PJ, Armstrong T (1994) Risk-taking by female ducks: intra- and interspecific tests of nest defense theory. Behav Ecol Sociobiol 34:79–85. 10.1007/BF00164178

[CR22] Frid A, Dill L (2002) Human-caused disturbance stimuli as a form of predation risk. Conserv Ecol 6(1):11. 10.5751/ES-00404-060111

[CR23] Gaillard J-M, Yoccoz NG (2003) Temporal variation in survival of mammals: a case of environmental canalization? Ecology 84:3294–3306. 10.1890/02-0409

[CR24] Gibelli J, Aubin-Horth N, Dubois F (2019) Individual differences in anxiety are related to differences in learning performance and cognitive style. Anim Behav 157:121–128. 10.1016/j.anbehav.2019.09.008

[CR25] Grieco F, van Noordwijk AJ, Visser ME (2002) Evidence for the effect of learning on timing of reproduction in blue tits. Science 296:136–138. 10.1126/science.1068287 11935025 10.1126/science.1068287

[CR26] Hanssen SA, Erikstad KE (2013) The long-term consequences of egg predation. Behav Ecol 24:564–569. 10.1093/beheco/ars198

[CR27] Hermansson I, von Numers M, Jaatinen K et al (2026) Navigating danger: how environmental cues and individual traits shape breeding dispersal in the endangered common eider. Behav Ecol. 10.1093/beheco/araf159

[CR28] Ims RA (1990) On the adaptive value of reproductive synchrony as a predator-swamping strategy. Am Nat 136:485–498. 10.1086/285109

[CR29] Jaatinen K, Öst M (2016) Brain size-related breeding strategies in a seabird. Oecologia 180:67–76. 10.1007/s00442-015-3468-2 26456024 10.1007/s00442-015-3468-2

[CR30] Jaatinen K, Møller AP, Öst M (2019) Annual variation in predation risk is related to the direction of selection for brain size in the wild. Sci Rep 9:11847. 10.1038/s41598-019-48153-w 31413345 10.1038/s41598-019-48153-w PMC6694153

[CR31] Jaatinen K, Westerbom M, Norkko A et al (2021) Detrimental impacts of climate change may be exacerbated by density‐dependent population regulation in blue mussels. J Anim Ecol 90:562–573. 10.1111/1365-2656.13377 33073861 10.1111/1365-2656.13377

[CR32] Jaatinen K, Hermansson I, Mohring B et al (2022) Mitigating impacts of invasive alien predators on an endangered sea duck amidst high native predation pressure. Oecologia 198:543–552. 10.1007/s00442-021-05101-8 35028754 10.1007/s00442-021-05101-8

[CR33] Jolles JW, Briggs HD, Araya-Ajoy YG, Boogert NJ (2019) Personality, plasticity and predictability in sticklebacks: bold fish are less plastic and more predictable than shy fish. Anim Behav 154:193–202. 10.1016/j.anbehav.2019.06.022

[CR34] Jones KA, Godin J-GJ (2010) Are fast explorers slow reactors? Linking personality type and anti-predator behaviour. Proc R Soc B 277:625–632. 10.1098/rspb.2009.1607 19864291 10.1098/rspb.2009.1607 PMC2842688

[CR35] Keogan K, Daunt F, Wanless S et al (2018) Global phenological insensitivity to shifting ocean temperatures among seabirds. Nat Clim Change 8:313–318. 10.1038/s41558-018-0115-z

[CR36] Kilpi M, Lindström K (1997) Habitat-specific clutch size and cost of incubation in common eiders, *Somateria mollissima*. Oecologia 111:297–301. 10.1007/s004420050238 28308122 10.1007/s004420050238

[CR37] Korschgen CE (1977) Breeding stress of female eiders in Maine. J Wildl Manag 41:360–373. 10.2307/3800505

[CR38] Kotrschal A, Rogell B, Bundsen A et al (2013) Artificial selection on relative brain size in the guppy reveals costs and benefits of evolving a larger brain. Curr Biol 23:168–171. 10.1016/j.cub.2012.11.058 23290552 10.1016/j.cub.2012.11.058 PMC3566478

[CR39] Kotrschal A, Corral-Lopez A, Amcoff M, Kolm N (2015) A larger brain confers a benefit in a spatial mate search learning task in male guppies. Behav Ecol 26:527–532. 10.1093/beheco/aru227 25825587 10.1093/beheco/aru227 PMC4374130

[CR40] Lane JE, Kruuk LEB, Charmantier A et al (2012) Delayed phenology and reduced fitness associated with climate change in a wild hibernator. Nature 489:554–557. 10.1038/nature11335 22878721 10.1038/nature11335

[CR41] Larsson K, Rattiste K, Lilleleht V (1997) Heritability of head size in the common gull *Larus canus* in relation to environmental conditions during offspring growth. Heredity 79:201–207. 10.1038/hdy.1997.143

[CR42] Lehikoinen A, Kilpi M, Öst M (2006) Winter climate affects subsequent breeding success of common eiders. Glob Change Biol 12:1355–1365. 10.1111/j.1365-2486.2006.01162.x

[CR43] Liedtke J, Fromhage L (2019) Modelling the evolution of cognitive styles. BMC Evol Biol 19(1):234. 10.1186/s12862-019-1565-2 31881934 10.1186/s12862-019-1565-2 PMC6935132

[CR44] Lima SL (2009) Predators and the breeding bird: behavioral and reproductive flexibility under the risk of predation. Biol Rev 84:485–513. 10.1111/j.1469-185X.2009.00085.x 19659887 10.1111/j.1469-185X.2009.00085.x

[CR45] Logan CJ, Kruuk LEB, Stanley R et al (2016) Endocranial volume is heritable and is associated with longevity and fitness in a wild mammal. R Soc Open Sci 3:160622. 10.1098/rsos.160622 28083105 10.1098/rsos.160622 PMC5210687

[CR46] Mathot KJ, Nicolaus M, Araya‐Ajoy YG et al (2015) Does metabolic rate predict risk‐taking behaviour? A field experiment in a wild passerine bird. Funct Ecol 29:239–249. 10.1111/1365-2435.12318

[CR47] Mohring B, Angelier F, Jaatinen K et al (2022) Drivers of within- and among-individual variation in risk-taking behaviour during reproduction in a long-lived bird. Proc R Soc B 289:20221338. 10.1098/rspb.2022.1338 36126681 10.1098/rspb.2022.1338 PMC9489283

[CR48] Mohring B, Öst M, Jaatinen K et al (2023) Breeding under pressure: corticosterone is associated with reproductive investment under fluctuating predation risk in a long-lived sea duck. Funct Ecol 37:2868–2882. 10.1111/1365-2435.14435

[CR49] Mohring B, Angelier F, Jaatinen K et al (2025) Habituation or sensitization? Short-term adjustment of flight initiation distance in incubating common eiders. Anim Behav 219:123030. 10.1016/j.anbehav.2024.11.008

[CR50] Öst M, Bäck A (2003) Spatial structure and parental aggression in eider broods. Anim Behav 66:1069–1075. 10.1006/anbe.2003.2300

[CR51] Öst M, Jaatinen K (2015) Smart and safe? Antipredator behavior and breeding success are related to head size in a wild bird. Behav Ecol 26:1371–1378. 10.1093/beheco/arv093

[CR52] Öst M, Steele BB (2010) Age-specific nest-site preference and success in eiders. Oecologia 162:59–69. 10.1007/s00442-009-1444-4 19727828 10.1007/s00442-009-1444-4

[CR53] Öst M, Wickman M, Matulionis E, Steele B (2008) Habitat-specific clutch size and cost of incubation in eiders reconsidered. Oecologia 158:205–216. 10.1007/s00442-008-1139-2 18795336 10.1007/s00442-008-1139-2

[CR54] Öst M, Lehikoinen A, Jaatinen K, Kilpi M (2011) Causes and consequences of fine-scale breeding dispersal in a female-philopatric species. Oecologia 166:327–336. 10.1007/s00442-010-1855-2 21120667 10.1007/s00442-010-1855-2

[CR55] Öst M, Lindén A, Karell P et al (2018) To breed or not to breed: drivers of intermittent breeding in a seabird under increasing predation risk and male bias. Oecologia 188:129–138. 10.1007/s00442-018-4176-5 29858692 10.1007/s00442-018-4176-5

[CR56] Öst M, Lehikoinen A, Jaatinen K (2022) Top-down effects override climate forcing on reproductive success in a declining sea duck. Oikos 2022:e08762. 10.1111/oik.08762

[CR57] Quinn JL, Cole EF, Bates J et al (2012) Personality predicts individual responsiveness to the risks of starvation and predation. Proc R Soc B 279:1919–1926. 10.1098/rspb.2011.2227 22179807 10.1098/rspb.2011.2227 PMC3311888

[CR58] R Core Team (2023) R: A language and environment for statistical computing. R Foundation for Statistical Computing. Austria: Vienna

[CR59] Réale D, Garant D, Humphries MM et al (2010) Personality and the emergence of the pace-of-life syndrome concept at the population level. Phil Trans R Soc B 365:4051–4063. 10.1098/rstb.2010.0208 21078657 10.1098/rstb.2010.0208 PMC2992747

[CR60] Rutberg AT (1987) Adaptive hypotheses of birth synchrony in ruminants: an interspecific test. Am Nat 130:692–710. 10.1086/284739

[CR61] Seltmann MW, Öst M, Jaatinen K et al (2012) Stress responsiveness, age and body condition interactively affect flight initiation distance in breeding female eiders. Anim Behav 84:889–896. 10.1016/j.anbehav.2012.07.012

[CR62] Sih A, Del Giudice M (2012) Linking behavioural syndromes and cognition: a behavioural ecology perspective. Phil Trans R Soc B 367:2762–2772. 10.1098/rstb.2012.0216 22927575 10.1098/rstb.2012.0216 PMC3427552

[CR63] Smaers JB, Rothman RS, Hudson DR et al (2021) The evolution of mammalian brain size. Sci Adv 7(18):2101. 10.1126/sciadv.abe2101 10.1126/sciadv.abe2101 PMC8081360 33910907

[CR64] Sommer-Trembo C, Zimmer C, Jourdan J et al (2016) Predator experience homogenizes consistent individual differences in predator avoidance. J Ethol 34:155–165. 10.1007/s10164-016-0460-1

[CR65] Stankowich T, Blumstein DT (2005) Fear in animals: a meta-analysis and review of risk assessment. Proc R Soc B 272:2627–2634. 10.1098/rspb.2005.3251 16321785 10.1098/rspb.2005.3251 PMC1559976

[CR66] Stoffel M, Nakagawa S, Schielzeth H, Goslee S (2017) rptR: repeatability estimation and variance decomposition by generalized linear mixed-effects models. Methods Ecol Evol 8:1639–1644. 10.1111/2041-210X.12797

[CR67] Swennen C (1990) Dispersal and migratory movements of eiders *Somateria mollissima* breeding in The Netherlands. Ornis Scand 21:17. 10.2307/3676374

[CR68] Switzer PV (1993) Site fidelity in predictable and unpredictable habitats. Evol Ecol 7:533–555. 10.1007/bf01237820

[CR69] van de Pol M, Wright J (2009) A simple method for distinguishing within- versus between-subject effects using mixed models. Anim Behav 77:753–758. 10.1016/j.anbehav.2008.11.006

[CR70] Verhulst S, Nilsson J-Å (2008) The timing of birds’ breeding seasons: a review of experiments that manipulated timing of breeding. Phil Trans R Soc B 363:399–410. 10.1098/rstb.2007.2146 17666390 10.1098/rstb.2007.2146 PMC2606757

[CR71] Wood KA, Thorstensen S, Lúðvíksson SJ et al (2020) Long‐term trends in the survival rates of adult female Common Eider *Somateria mollissima* at three colonies in Iceland. Ibis. 10.1111/ibi.12893

[CR72] Zanette LY, White AF, Allen MC, Clinchy M (2011) Perceived predation risk reduces the number of offspring songbirds produce per year. Science 334:1398–1401. 10.1126/science.1210908 22158817 10.1126/science.1210908

[CR73] Zettlemoyer MA, DeMarche ML (2022) Dissecting impacts of phenological shifts for performance across biological scales. Trends Ecol Evol 37:147–157. 10.1016/j.tree.2021.10.004 34763943 10.1016/j.tree.2021.10.004

