# Supplementary material for: Predation risk is associated with head-size-related divergence in breeding phenology in a female sea duck
Source: Oecologia. 2026 Apr 24;208(5):61. doi: 10.1007/s00442-026-05891-9 (PMC13109241; doi:10.1007/s00442-026-05891-9)
Supplement: Supplementary file 1 — Supplementary file1 (DOCX 444 KB) [file 442_2026_5891_MOESM1_ESM.docx]

**Supplementary Information**

for

**Predation risk is associated with head-size-related divergence in breeding phenology in a female sea duck**

**Bertille Mohring**^*+^, **Ida Hermansson, Kim Jaatinen, Markus Öst**^1*+^

**^*^**Corresponding authors: [bmohring@liverpool.ac.uk](mailto:bmohring@liverpool.ac.uk) and [markus.ost@abo.fi](mailto:markus.ost@abo.fi)

**^+^**These authors contributed equally to this work

**Supplementary Figure S1:** Relationship between female common eider relative laying date and the two-way interactions between (A) island-specific nest predation risk and relative head volume, (B) island-specific adult predation risk and relative head volume, (C) island-specific nest predation risk and median lifetime FID and (D) island-specific adult predation risk and median lifetime FID. The solid line denotes females with (A, B) a large relative head volume or (C, D) a long FID (mean + 1 SD), the dashed line females with (A, B) an intermediate relative head volume or (C, D) an intermediate FID (mean) and the dotted line females with (A, B) a small relative head volume or (C, D) a short FID (mean - 1 SD). Areas account for 95% confidence intervals.

**
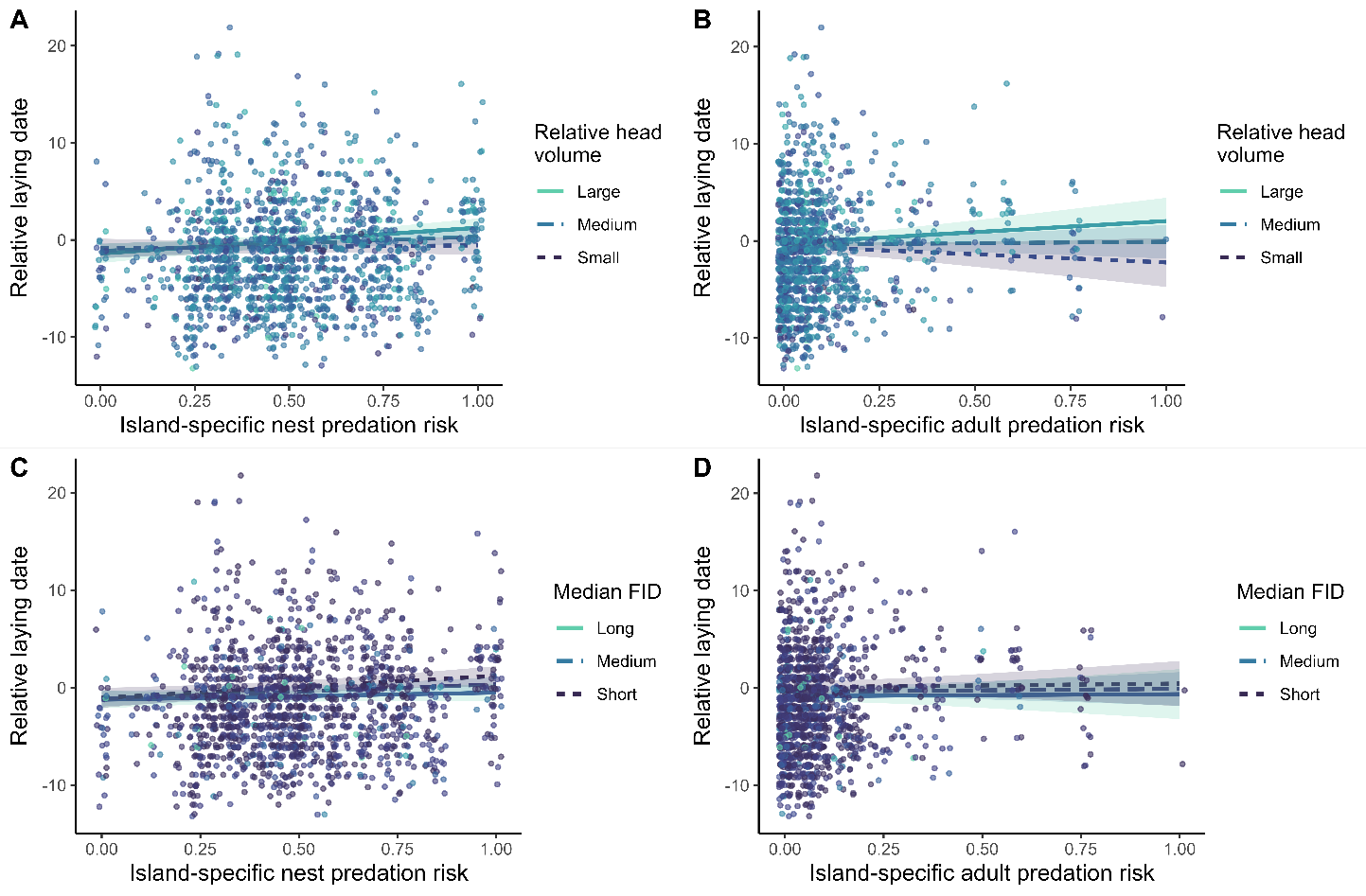
**

**Supplementary Figure S2:** Correlation plot of the continuous explanatory variables tested in the two linear mixed models. The variables include relative date of nest visit, female median lifetime FID, island-specific nest predation risk, island-specific adult predation risk, female relative head volume and female body condition.

**
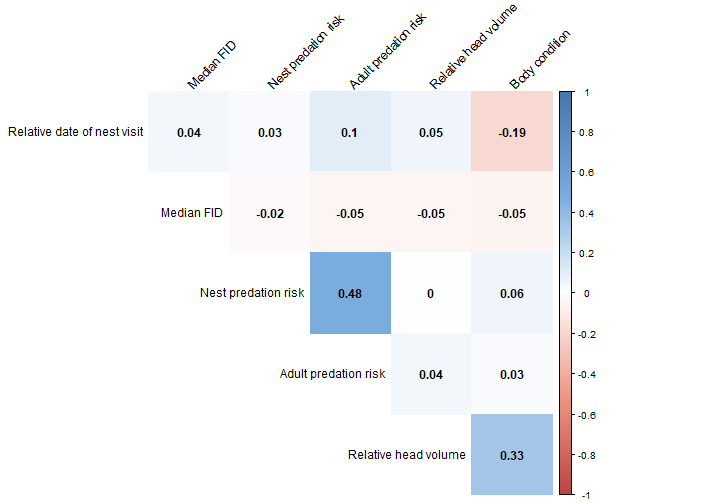
**

**Supplementary Table S1:** Table presenting the top-ranked linear mixed models within ΔAICc ≤ 10 explaining variation in female common eider relative laying date in relation to individual characteristics (breeding experience, relative head volume, median lifetime FID), relative date of nest visit, nest or adult predation risk (respectively) and the two-way interactions between the predation risk indices (nest or adult predation risk, respectively) and female relative head volume and between the predation risk indices (nest or adult predation risk, respectively) and median lifetime FID. Female and island identity were included as random intercept effects in all models.

|  | **Fixed effect** | **df^1^** | **log-lik^2^** | **AICc** | **ΔAICc** | **w_i_^3^** | **R²_m_^4^** | **R²_c_^5^** |
| --- | --- | --- | --- | --- | --- | --- | --- | --- |
|  | **Nest predation risk model** |  |  |  |  |  |  |  |
| M_1_ | Breed exp + RHV + FID + Nest pred + Date + Nest pred × RHV + Nest pred × FID | 11 | -4226.01 | 8474.20 | 0.00 | 0.26 | 0.22 | 0.38 |
| M_2_ | Breed exp + RHV + FID + Nest pred + Date + Nest pred × RHV | 10 | -4227.18 | 8474.52 | 0.32 | 0.22 | 0.22 | 0.38 |
| M_3_ | Breed exp + BC + RHV + FID + Nest pred + Date + Nest pred × RHV + Nest pred × FID | 12 | -4225.60 | 8475.41 | 1.21 | 0.14 | 0.23 | 0.39 |
| M_4_ | Breed exp + BC + RHV + FID + Nest pred + Date + Nest pred × RHV | 11 | -4226.80 | 8475.77 | 1.57 | 0.12 | 0.22 | 0.38 |
| M_5_ | Breed exp + RHV + FID + Nest pred + Date + Nest pred × FID | 10 | -4228.33 | 8476.81 | 2.61 | 0.07 | 0.22 | 0.38 |
| M_6_ | Breed exp + RHV + FID + Nest pred + Date | 9 | -4229.83 | 8477.77 | 3.57 | 0.04 | 0.22 | 0.38 |
| M_7_ | Breed exp + BC + RHV + FID + Nest pred + Date + Nest pred × FID | 11 | -4227.93 | 8478.05 | 3.85 | 0.04 | 0.22 | 0.38 |
| M_8_ | Breed exp + FID + Nest pred + Date + Nest pred × FID | 9 | -4230.09 | 8478.31 | 4.10 | 0.03 | 0.22 | 0.38 |
| M_9_ | Breed exp + BC + RHV + FID + Nest pred + Date | 10 | -4229.46 | 8479.06 | 4.86 | 0.02 | 0.22 | 0.38 |
| M_10_ | Breed exp + FID + Nest pred + Date | 8 | -4231.63 | 8479.37 | 5.16 | 0.02 | 0.22 | 0.38 |
| M_11_ | Breed exp + BC + FID + Nest pred + Date + Nest pred × FID | 10 | -4230.05 | 8480.26 | 6.05 | 0.01 | 0.22 | 0.38 |
| M_12_ | Breed exp + BC + FID + Nest pred + Date | 9 | -4231.60 | 8481.33 | 7.13 | 0.01 | 0.22 | 0.38 |
| M_13_ | Breed exp + RHV + FID + Date | 8 | -4232.98 | 8482.07 | 7.86 | 0.01 | 0.22 | 0.38 |
| M_14_ | Breed exp + RHV + Nest pred + Date + Nest pred × RHV | 9 | -4232.06 | 8482.25 | 8.05 | 0.00 | 0.22 | 0.39 |
| M_15_ | Breed exp + BC + RHV + Nest pred + Date + Nest pred × RHV | 10 | -4231.62 | 8483.39 | 9.18 | 0.00 | 0.22 | 0.39 |
| M_16_ | Breed exp + BC + RHV + FID + Date | 9 | -4232.76 | 8483.64 | 9.44 | 0.00 | 0.22 | 0.38 |
| M_17_ | Breed exp + FID + Date | 7 | -4234.78 | 8483.64 | 9.44 | 0.00 | 0.21 | 0.37 |
|  | **Adult predation risk model** |  |  |  |  |  |  |  |
| M_1_ | Breed exp + RHV + FID + Ad pred + Date + Ad pred × RHV | 10 | -4230.73 | 8481.61 | 0.00 | 0.21 | 0.22 | 0.38 |
| M_2_ | Breed exp + RHV + FID + Date | 8 | -4232.98 | 8482.07 | 0.46 | 0.17 | 0.22 | 0.38 |
| M_3_ | Breed exp + BC + RHV + FID + Ad pred + Date + Ad pred × RHV | 11 | -4230.46 | 8483.11 | 1.50 | 0.10 | 0.22 | 0.38 |
| M_4_ | Breed exp + RHV + FID + Ad pred + Date + Ad pred × RHV + Ad pred × FID | 11 | -4230.72 | 8483.63 | 2.02 | 0.08 | 0.22 | 0.38 |
| M_5_ | Breed exp + BC + RHV + FID + Date | 9 | -4232.76 | 8483.64 | 2.03 | 0.08 | 0.22 | 0.38 |
| M_6_ | Breed exp + FID + Date | 7 | -4234.78 | 8483.64 | 2.04 | 0.08 | 0.21 | 0.37 |
| M_7_ | Breed exp + RHV + FID + Ad pred + Date | 9 | -4232.87 | 8483.86 | 2.26 | 0.07 | 0.22 | 0.38 |
| M_8_ | Breed exp + BC + RHV + FID + Ad pred + Date + Ad pred × RHV + Ad pred × FID | 12 | -4230.46 | 8485.14 | 3.53 | 0.04 | 0.22 | 0.38 |
| M_9_ | Breed exp + FID + Ad pred + Date | 8 | -4234.65 | 8485.40 | 3.79 | 0.03 | 0.21 | 0.37 |
| M_10_ | Breed exp + BC + RHV + FID + Ad pred + Date | 10 | -4232.64 | 8485.43 | 3.83 | 0.03 | 0.22 | 0.38 |
| M_11_ | Breed exp + BC + FID + Date | 8 | -4234.78 | 8485.66 | 4.05 | 0.03 | 0.21 | 0.37 |
| M_12_ | Breed exp + RHV + FID + Ad pred + Date + Ad pred × FID | 10 | -4232.83 | 8485.81 | 4.21 | 0.03 | 0.21 | 0.37 |
| M_13_ | Breed exp + FID + Ad pred + Date + Ad pred × FID | 9 | -4234.61 | 8487.35 | 5.75 | 0.01 | 0.22 | 0.38 |
| M_14_ | Breed exp + BC + RHV + FID + Ad pred + Date + Ad pred × FID | 11 | -4232.61 | 8487.39 | 5.79 | 0.01 | 0.21 | 0.37 |
| M_15_ | Breed exp + BC + FID + Ad pred + Date | 9 | -4234.65 | 8487.42 | 5.81 | 0.01 | 0.22 | 0.38 |
| M_16_ | Breed exp + BC + FID + Ad pred + Date + Ad pred × FID | 10 | -4234.61 | 8489.38 | 7.77 | 0.00 | 0.21 | 0.37 |
| M_17_ | Breed exp + RHV + Ad pred + Date + Ad pred × RHV | 9 | -4235.84 | 8489.80 | 8.20 | 0.00 | 0.21 | 0.37 |
| M_18_ | Breed exp + RHV + Date | 7 | -4238.05 | 8490.18 | 8.57 | 0.00 | 0.21 | 0.38 |
| M_19_ | Breed exp + BC + RHV + Ad pred + Date + Ad pred × RHV | 10 | -4235.53 | 8491.21 | 9.61 | 0.00 | 0.21 | 0.38 |

Model term abbreviations: ‘Breed Exp’: female minimum breeding experience; ‘BC’: female body condition; ‘RHV’: female relative head volume; ‘FID’: female lifetime median FID; ‘Nest pred’: island-specific nest predation risk; ‘Ad pred’: island-specific adult predation risk; ‘Date’: relative date of nest visit.

^1^ df: degrees of freedom

^2^ log-lik: log-likelihood

^3^ w_i_: Akaike weight

^4^ R²_m_: marginal R², representing the variance explained by fixed factors

^5^ R²_c_: conditional R², representing the variance explained by both fixed and random effects

**Supplementary Table S2:** Model-averaged coefficient estimates (E), standard errors (SE), z-values and p-values derived from the top-ranked linear mixed models (LMMs) within ΔAICc ≤10 explaining variation in female common eider relative laying date in relation to individual characteristics (breeding experience, relative head volume, median lifetime FID), relative date of nest visit, nest or adult predation risk (respectively) and the two-way interactions between the predation risk indices (nest or adult predation risk, respectively) and female relative head volume and between the predation risk indices (nest or adult predation risk, respectively) and median lifetime FID. Female and island identity were included as random intercept effects in all models. Significant effects (*p* ≤ 0.05) are presented in bold and nearly significant effects are presented in bold and italics (*p* ≤ 0.1).

| **Fixed effect** | **E ± SE** | **z-value** | **p-value** |
| --- | --- | --- | --- |
| **Conditional model averaging on nest predation risk model** |  |  |  |
| Intercept | -0.37 ± 0.27 | 1.36 | 0.173 |
| **Breeding experience: Experienced** | **-1.69 ± 0.25** | **6.66** | **<0.001** |
| Body condition | -0.12 ± 0.14 | 0.85 | 0.397 |
| ***Relative head volume*** | ***0.29 ± 0.15*** | ***1.95*** | ***0.051*** |
| **Median lifetime FID** | **-0.47 ± 0.14** | **3.25** | **0.001** |
| **Nest predation risk** | **0.31 ± 0.12** | **2.58** | **0.010** |
| **Relative date of nest visit** | **2.13 ± 0.12** | **17.40** | **<0.001** |
| **Relative head volume × nest predation risk** | **0.26 ± 0.11** | **2.23** | **0.026** |
| Median FID × nest predation risk | -0.17 ± 0.11 | 1.59 | 0.112 |
| **Conditional model averaging on adult predation risk model** |  |  |  |
| Intercept | -0.34 ± 0.28 | 1.20 | 0.231 |
| **Breeding experience: Experienced** | **-1.71 ± 0.25** | **6.73** | **<0.001** |
| Body condition | -0.08 ± 0.14 | 0.60 | 0.549 |
| ***Relative head volume*** | ***0.29 ± 0.15*** | ***1.96*** | ***0.050*** |
| **Median lifetime FID** | **-0.47 ± 0.14** | **3.24** | **0.001** |
| **Adult predation risk** | 0.05 ± 0.12 | 0.41 | 0.684 |
| **Relative date of nest visit** | **2.13 ± 0.12** | **17.41** | **<0.001** |
| **Relative head volume × adult predation risk** | **0.26 ± 0.13** | **2.08** | **0.038** |
| Median FID × adult predation risk | -0.02 ± 0.13 | 0.16 | 0.877 |

**Supplementary Table S3:** LMMs explaining female common eider relative laying date in relation to individual characteristics (breeding experience, relative head volume), relative date of nest visit, nest (M_1_) or adult (M_2_) predation risk, and flight initiation distance (FID). FID was decomposed via within-individual centring into a between-individual component (FID_between_; individual mean) and a within-individual component (FID_within_; annual deviation from the individual mean). Models also included separate two-way interactions between predation risk (nest predation risk in M_1_; adult predation risk in M_2_) and (i) female relative head volume, (ii) FID_between_, and (iii) FID_within_. Female and island identity were included as random effects. As these analyses require year-specific FID measurements, the data were restricted to 954 breeding attempts by 473 females with FID recorded in the focal year. Significant effects (*p* ≤ 0.05) are presented in bold, and trends (0.05 < *p* ≤ 0.10) in bold italics.

| **Fixed effect** | **Mean estimate (E) ± SE** | **t-value** | **p-value** |
| --- | --- | --- | --- |
| **M_1_: Nest predation risk model** |  |  |  |
| **Intercept** | **-1.42 ± 0.26** | **-5.55** | **<0.001** |
| **Breeding experience: Experienced** | **-1.48 ± 0.26** | **-5.75** | **<0.001** |
| Body condition | 0.20 ± 0.14 | 1.47 | 0.143 |
| Relative head volume | 0.18 ± 0.14 | 1.24 | 0.216 |
| FID_within_ | 0.13 ± 0.11 | 1.18 | 0.238 |
| **FID_between_** | **-0.46 ± 0.13** | **-3.45** | **<0.001** |
| Nest predation risk | 0.10 ± 0.12 | 0.83 | 0.407 |
| **Relative date of nest visit** | **2.26 ± 0.13** | **17.85** | **<0.001** |
| Relative head volume × nest predation risk | 0.07 ± 0.12 | 0.62 | 0.535 |
| FID_within_ × nest predation risk | -0.12 ± 0.10 | -1.15 | 0.252 |
| FID_between_ × nest predation risk | -0.04 ± 0.10 | -0.41 | 0.681 |
| **M_2_: Adult predation risk model** |  |  |  |
| Intercept | **-1.42 ± 0.26** | **-5.51** | **<0.001** |
| **Breeding experience: Experienced** | **-1.50 ± 0.26** | **-5.83** | **<0.001** |
| Body condition | 0.21 ± 0.14 | 1.55 | 0.122 |
| **Relative head volume** | 0.19 ± 0.14 | 1.35 | 0.177 |
| FID_within_ | 0.12 ± 0.11 | 1.17 | 0.244 |
| **FID_between_** | **-0.46 ± 0.14** | **-3.40** | **<0.001** |
| Adult predation risk | 0.01 ± 0.12 | 0.09 | 0.925 |
| **Relative date of nest visit** | **2.26 ± 0.13** | **17.89** | **<0.001** |
| **Relative head volume × adult predation risk** | ***-0.24 ± 0.13*** | ***1.80*** | ***0.073*** |
| FID_within_ × adult predation risk | -0.17 ± 0.11 | -1.49 | 0.137 |
| FID_between_ × adult predation risk | 0.01 ± 0.14 | -0.06 | 0.947 |
